# Association between hepatitis B virus infection and metabolic syndrome: a retrospective cohort study in Shanghai, China

**DOI:** 10.1186/1471-2458-14-516

**Published:** 2014-05-28

**Authors:** Yanbing Zhou, Yan Cui, Haiju Deng, Jinming Yu

**Affiliations:** 1 Center for Infection and Immunity, School of Public Health, Columbia University, New York City, USA; 2 Shanghai Centers for Disease Control and Prevention, Putuo Branch, Shanghai, P.R. China; 3 School of Public Health, Fudan University, Shanghai, P.R. China

## Abstract

**Background:**

Metabolic syndrome (MS) and hepatitis B (HBV) infection are two major public health problems in China. There are few studies about their association, and the results of these studies are contradictory. We conducted a retrospective cohort study to assess the association between MS and HBV in a Shanghai community-based cohort.

**Methods:**

Nine hundred seventy-six Shanghai residents were recruited from the Putuo community. 480 HBV infections were in exposed group and 496 non-infections in unexposed group. All metabolic-related parameters and hepatitis B serology were tested with routine biochemical or immunological methods. “Exposed” was defined by HBV infection represented by hepatitis B surface antigen (HBsAg) and without anti-virus treatment. “Unexposed” were subjects who didn’t infect with HBV (Represented by HBsAg) and no MS when they entered the cohort. MS was defined based on the updated National Cholesterol Education Program Adult Treatment Panel III criteria. The Cox proportional hazards model was used to estimate the hazard ratios (HR) and related 95% confidence intervals (95% CI) for the association between HBV infection and MS over a 20-year follow-up period.

**Results:**

Of 976 subjects recruited, 480 had latent HBV infection (exposed subjects). After adjusting for age, the crude HR was 2.46 (95% CI: 1.77, 3.41). After adjusting for potential risk factors of MS (age, gender, smoking, passive smoking, alcohol consumption, physical activity, and diet), the HR was 2.27 (95% CI: 1.52, 3.38).

**Conclusions:**

This 20-year follow-up retrospective cohort study in Shanghai showed a positive association between HBV infection and MS.

## Background

Metabolic syndrome (MS) is a complicated metabolic disorder comprising obesity, hypertension, diabetes, and dyslipidemia. MS has been verified as a risk factor for cardiovascular disease, type 2 diabetes, and renal disease [1-5], and increases mortality from all causes [6]. MS is recognized as a major public health problem worldwide [7,8].

China is endemic for hepatitis B virus (HBV) infection, with a prevalence of nearly 10% [9] in those subjects over 40 in 2006. This age cohort did not receive regular hepatitis B vaccination.

Evidence suggests that hepatitis C virus (HCV) infection has an impact on lipid and glucose metabolism [10-14]. A community-based study in Taiwan found HCV infection to be associated with MS (odds ratio 6.4; 95% CI: 1.82 to 22.84) [15]. Similarly to HCV, it is thought that HBV might lead to chronic liver damage by dyslipidemia. A study by Su *et al.*[16] reported an association between asymptomatic chronic HBV infection and lower serum levels of total cholesterol (TC) and high-density lipoprotein cholesterol (HDL-C). This evidence suggests that HBV might affect metabolic profiles and subsequent development of MS. However, studies of the correlation between HBV infection and MS yield contradictory results. Three studies reported a positive association between HBV infection and MS [17-19], two found an inverse association [20,21], and one found no association [22]. The contradictions may be due to small sample size [23] and limitation of subjects to hospital patients [24]. Another study suggested that MS might cause worsening of cirrhosis in chronic HBV patients [25], suggesting a relationship between MS and HBV infection. It is vital to explore the relationship between two of the major public health problems of China, HBV and MS, to inform prevention and control strategies.

## Methods

### Population and study design

**Figure 1:**
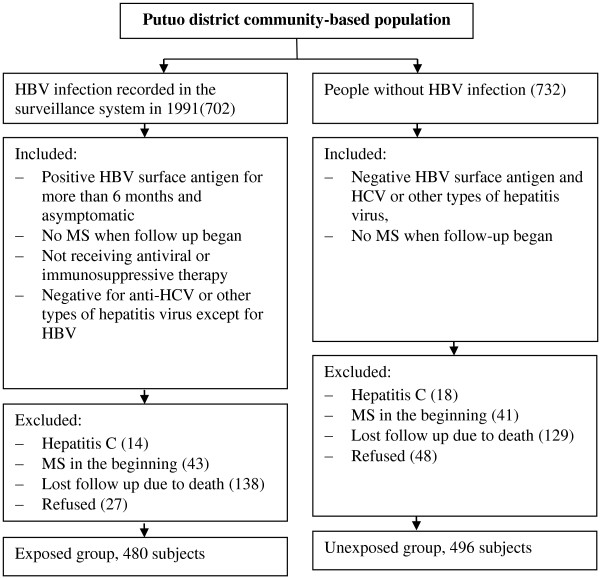
Selection of subjects during study.

This study was conducted with the approval of the Ethical Review Board in the School of Public Health, Fudan University and conforms to the principles embodied in the *Declaration of Helsinki*.We evaluated the association between MS and HBV in a community-based retrospective study founded in 1991 in Shanghai. The cohort was from Putuo district, located in the northwest of Shanghai, China. Subjects with HBV were recruited via records from 1991 from the population-based infectious disease surveillance system. Subjects with latent HBV infections (defined as asymptomatic, but positive for HBV surface antigen for more than 6 months) were classified as “exposed” (Figure 1). After excluding subjects with MS when they entered into the cohort, subjects who had received antiviral or immunosuppressive therapy, and subjects without informed consent, 480 subjects were enrolled into the study. All of them were negative for anti-HCV and other types of hepatitis virus, except for HBV.Four hundred ninety-six subjects negative for HBV surface antigen from the same community were classified as “unexposed”. We excluded subjects positive for HCV or other types of hepatitis virus, as well as those who had MS when they entered into the cohort (Figure 1).

For each subject, demographic characteristics, family history, information about tobacco smoking, alcohol consumption, physical activity, and diet were collected using a questionnaire on 31 December 2011, the day the study was completed. Data from physical examinations and medical histories were collected at entry into the study from hospital records.

Subjects were followed until MS (the endpoint) appeared or until study completion. Time of follow-up (in person-years) was calculated for each subject as the difference between the date of entry into the cohort and date of endpoint or date of the end of the study period, whichever came first. All subjects were given a physical examination at the end of the study.

Physical examination variables measured were the following: waist circumference measured at the midpoint between the lower border of the rib cage and the iliac crest; seated blood pressure measured 3 times, 1 min apart, using a standard mercury sphygmomanometer, with participants resting for at least 30 minutes before the measurements were made. Blood samples were collected by venipuncture in the morning after 12 hours fasting. Laboratory variables measured from the blood samples were triglycerides (TG), low-density lipoprotein cholesterol (LDL-C), HDL-C, TC, fasting plasma glucose (FPG), and hepatitis B serology. Definitions of smoking status for subjects were current smokers (if they were actively smoking) and previous smokers (if they had quit smoking for the past 6 months). Definitions of passive smoking status for subjects reported their history of passive smoking exposure longer than 5 years. Definitions of alcohol consumption status for subjects were current alcohol consumption (if they were actively drinking regardless of amount) and previous smokers (if they had quit drinking for the past 6 months). Definitions of high-energy food intake were subjects who reported that they took fried, smoked, pickled foods and sweets over 5 times in one week. Definitions of fresh fruits and vegetables intake were subjects who reported that they took fruits and fresh vegetables over 5 times in one week. Physical activity was divided into 2 levels, none physical activity was defined as those who exercised less than 1 hour per week.

### Definition of metabolic syndrome

MS was diagnosed based on the updated National Cholesterol Education Program Adult Treatment Panel III criteria [4], requiring the presence of three or more of the following five criteria: (1) waist circumference >90 cm in men or >80 cm in women; (2) TG >150 mg/dL; (3) HDL-C <40 mg/dL in men or <50 mg/dL in women; (4) blood pressure >130/85 mmHg or current use of antihypertensive medications; (5) FPG >100 mg/dL or use of oral anti-diabetic agents or insulin.

As a retrospective cohort study, all necessary data was tracked and collected in 2012 via database/ medical record. In this type study, we need a good standard to determine disease status at present time though the standard didn’t exist in past, so we selected ATP III as the definition for metabolic syndrome to evaluate the association between metabolic syndrome and hepatitis B infection.

### Serum viral markers and biochemistry

Blood samples were tested in the laboratory of the Putuo District Hospital. TG, LDL-C, HDL-C, TC and FPG were measured using Shanghai standard laboratory test criteria. HBsAg was detected using routine standard ELISA.

### Statistical analysis

The data were presented as percentages for categorical variables as well as mean with standard deviations for continuous variables unless mentioned otherwise. Appropriate comparison tests including Chi-square test and Student’s *t* test were used for comparison between groups for categorical variables and continuous variables, respectively.

To examine the independent associations between potential risk factors and MS, we conducted survival analysis using Cox proportional hazards models. The covariates in our models included factors that might influence the development of MS. The strength of association was presented as hazards ratio (HR) with 95% confidence intervals (95% CI) and P-values. Analysis was performed using the SAS 9.2 statistical package. All tests were two-sided and p < 0.05 was considered statistically significant. First we carried out crude HR calculation for each potential risk factor adjusting for common confounders such as age, then explored the association between HBV infection and MS by adjusting all potential risk factors.

## Results

### Baseline demographic features

Of the 976 subjects recruited for this study, 480 were classified as exposed (with latent HBV infection), and 496 were classified as unexposed. The 591 male and 385 female subjects had a median age of 55.5 years. Mean follow-up was 20.8 person-years in the exposed group and 20.6 person-years in the unexposed group. Table 1 lists characteristics of both cohorts. Gender, age, education, race, and family income of the two cohorts were similar; the cohorts differed in occupation and marital status (Chi-square test, p > 0.05; see Table 1).

**Table 1:** Comparison of baseline characteristics between subjects with latent HBV infection (exposed) and the unexposed group

**Baseline characteristics**	**Exposed group**	**Unexposed group**	**P-value**
	**(N = 480)**	**(N = 496)**	
**Gender**			
Male	295 (61.5%)	296 (59.7%)	0.5693
**Age (years)**			
<35	17 (3.5%)	16 (3.2%)	0.3498
35-44	59 (12.3%)	45 (9.1%)	
45-54	161 (33.5%)	168 (33.9%)	
55-64	165 (34.3%)	192 (38.7%)	
≥65	78 (16.4%)	75 (15.1%)	
**Occupation**			
Factory worker	110 (22.9%)	80 (16.1%)	0.0274
Light manual worker	298 (59.9%)	337 (67.9%)	
Catering staff	72 (17.2%)	79 (16.0%)	
**Education**			
Primary school	41 (8.5%)	42 (8.5%)	0.0581
Middle school	358 (74.6%)	399 (80.4%)	
College and above	81 (16.9%)	55 (11.1%)	
**Race**			
Ethnic Han	471 (98.1%)	487 (98.2%)	0.9440
Others	9 (1.9%)	9 (1.8%)	
**Marital status**			
Never married	31 (6.5%)	12 (2.4%)	0.0087
Married	419 (87.3%)	450 (90.7%)	
Divorced	30 (6.2%)	34 (6.9%)	
**Family income (10,000 RMB)**			
<1	42 (8.8%)	31 (6.3%)	0.0917
1 - <3	125 (26.0%)	126 (25.4%)	
3 - <5	180 (37.5%)	190 (38.3%)	
5 - <10	113 (23.5%)	114 (23.0%)	
≥10	20 (4.2%)	35 (7.0%)	

### Analyses of possible risk factors

Table 2 shows the results of analyses of known risk factors for MS. There were no differences in the two groups for these factors (Chi-square test, p > 0.05).

**Table 2:** Comparison of potential risk factors between HBV infection exposed group and unexposed group

**Potential risk factors**	**Exposed group**	**Unexposed group**	**P-value**
	**(N = 480)**	**(N = 496)**	
**Smoking**			
Yes	184 (38.3%)	170 (34.3%)	0.1873
**Passive smoking**			
Yes	263 (54.8%)	253 (51.0%)	0.2365
**Alcohol consumption**			
Yes	133 (27.7%)	159 (32.1%)	0.1380
**High-energy food intake**			
Yes	192 (40.0%)	214 (43.1%)	0.3189
**Fresh fruit and vegetable intake**			
Yes	254 (52.9%)	267 (53.8%)	0.7748
**Physical activity**			
Yes	57 (11.9%)	68 (13.7%)	0.3912

### Association of latent HBV infection with metabolic syndrome

Until the end of the study period, the crude incidence of MS across the entire study population was 171 out of 976, and the average rate of MS was 85 per 10000 person-years. The crude incidence of MS was 119.4 in the exposed group and 50.8 in the unexposed group.

In the unadjusted analyses, the crude age-adjusted hazard ratio between HBV infection and MS was 2.46 (95% CI: 1.77, 3.41).

To further clarify the association between latent HBV infection and MS, the potential risk factors of age, gender, smoking, alcohol consumption, physical activity, and diet were included in a multivariate Cox hazards ratio model. HBV infection and MS were still positively associated (HR, 2.27; 95% CI: 1.52, 3.38), after adjusting for these potential risks (Table 3).

**Table 3:** Event rates and hazards rations for exposed and unexposed groups

**Characteristics**	**Exposed group (HBV infection)**	**Unexposed group (No HBV infection)**
**No. (%) of incidence of MS**	119 (24.8)	52 (10.5)
**Mean (SD) duration of follow-up (years)**	20.8 (2.0)	20.6 (2.4)
Crude event rate (No. of events per 10000 person-years)*	119.4	50.8
**Unadjusted hazards ratio (95% CI) †**	2.46 (1.77 to 3.41)	1.00
**Adjusted hazards ratio (95% CI) ∆**	2.27 (1.52 to 3.38)	1.00

*(No. of events/total No. of person-years) × 10,000. **†** Adjusted for age; ∆ Adjusted for age; gender; smoking; passive smoking; alcohol consumption; high-energy food intake; fresh fruit and vegetable intake; physical activity.

## Discussion

In this retrospective cohort study, we enrolled 976 subjects, including 480 with latent HBV infections and 496 without HBV infections. Using univariate Cox hazards ratio model analysis, we estimated the age-adjusted hazards ratio between latent HBV infection and MS (HR, 2.46; 95% CI: 1.77, 3.41). Using multivariate Cox hazards ratio model analysis, we found that a positive association between latent HBV infection and MS (HR, 2.27; 95% CI: 1.52, 3.38) after adjusting for risk factors of MS (age, gender, smoking, passive smoking, alcohol consumption, physical activity, and diet). We concluded that this retrospective cohort study shows a positive association between latent HBV infection and MS in this cohort.

Two cross-sectional studies in Hangzhou and Taiwan reported decreased risk for MS in subjects with HBV infection [20,21]. A cross-section study in Hangzhou showed MS prevalence in subjects with chronic HBV to be 5.9% and 8.8% in the control group (OR = 0.65, 95% CI: 0.48, 0.88) [21]. The same result was observed in the Taiwan Keelung Community-based Integrated Screening study, MS prevalence in subjects with chronic HBV and control subjects was 8% and 10.9% respectively (OR = 0.84, 95% CI: 0.76, 0.93) [20]. In these studies, the criteria for defining MS were different from those of this study, and the prevalence of MS in HBV infected subjects may have been underestimated. Moreover, some potential confounders such as BMI and smoking were not adequately adjusted for. These studies were also limited by sample size and representation. The largest and latest study which included 593,594 subjects with chronic hepatitis B from NHANES III in US also reported an inverse relationship between chronic hepatitis B infection and MS [26]. This study was cross-section study design it included subjects from 2 months to elder. Two independent cross-section studies from Taiwan [27] and Slovakia [28] showed that there was no association between HBV and MS, in Taiwan’s study no individual components of MS was associated with HBV infection but opposite result found in Slovakia’s study that HBV infection may decrease level of TC and LDL. In other cross-section study from Taiwan showed that compared with healthy person, patients with chronic HBV infection had lower level of TG and LDL [29]. However these cross-section studies cannot examine the temporal association. In contrast, we used a retrospective cohort study based on a community population, and subjects with latent HBV infection received no antiviral therapy. Over an almost 20-year follow-up period, adjusted for some possible confounders, we evaluated the association between HBV infection and MS. Similarly, Yen *et al*. [30] found that, compared to subjects with serum protective titers from hepatitis B vaccination, those with natural HBV infection had 58% higher risk for MS after adjusting for age, gender, BMI, smoking and other possible confounders. A previous study indicated that insulin resistance with excessive flux of fatty acids is implicated as a possible mechanisms of MS [31]. There is a large body of evidence supporting a possible connection between HCV infection and MS through insulin resistance [15,32-34]. It is still unknown if there is a similar mechanism between HBV infection and MS. Further biological, molecular and genetic studies are needed to test this hypothesis.

A dramatic decline of HBV infection in Chinese children began more than two decades ago, due to an effective hepatitis B vaccination program [35]. Nevertheless, HBV infection is still a severe health threat to adults over 40 years old without regular hepatitis B vaccination, with the prevalence of HBV infection at nearly 10% [9]. MS has become one of the major public health challenges in China; the prevalence according to modified ATP III criteria in adult men was 28.4% and 35.1% in adult women, in a community-based survey in Shanghai [36]. Our study indicates that latent HBV infection is a risk factor for MS development. Thus, in planning the prevention and control strategy for MS, more attention must be given to addressing the needs of the population with ‘silent’ HBV.

Our study has several potential limitations. First, our study is observational, and although the baseline differences between our cohorts were minor, we may not have been able to adjust adequately for such differences. Important confounders may also have been unrecognized. Second, the detection methods, reference ranges and detection reagents for HBV infection varied over the 20-year period of the study. Third, we reviewed all recorded all medical history of subjects to define the endpoint of MS. We assumed that MS in this population would be represented in hospital records, even if all cases did not meet the same lab criteria. Fourth, given the limitations of our data, we could not adjust for all known important factors affecting the risk of MS, such as diet, presence and severity of hypertension, and lipid status. Fifth, inconsistent with other studies [37-39], we did not find associations between lifestyle factors such as smoking, alcohol consumption, high-energy food intake, fresh fruit and vegetable intake, physical activity and MS in this study. This is doubtless because of the difficulties in obtaining this type of detailed information for a retrospective study.

## Conclusions

Our study shows that latent HBV infection is associated with an increased prevalence of MS. It is critical that the medical establishment give more attention to those over 40 with have latent HBV infections, as they have increased risk for developing MS and will increase the chronic disease burden in China.

## Competing interests

All authors declare that they have no competing interests.

## Authors’ contributions

YBZ and JMY designed the study protocol; YBZ, YC and HJD conducted the questionnaire design, data collected and analyzed and manuscript drafted. All authors commented on and contributed to each version of the paper and approved the final version.

## Pre-publication history

The pre-publication history for this paper can be accessed here:

http://www.biomedcentral.com/1471-2458/14/516/prepub
